# Meeting clinical goals for the maintenance of the potential organ donor can reduce the loss of donors by cardiac arrest

**DOI:** 10.1186/cc14717

**Published:** 2015-09-28

**Authors:** Miriam Cristine V Machado, Artur Montemezzo, Fernanda Cani, Gabriel Torres, Glauco A Westphal, Joel de Andrade, Leandro Botelho, Silvana Wagner, Stefan Halla, Tiago C Carnin

**Affiliations:** 1 Central de Notificação, Captação e Distribuição de Órgãos do Estado de Santa Catarina, Florianópolis, SC, Brazil

## Introduction

The disproportion between the large organ demand and the low number of transplantations performed represents a serious public health problem worldwide. In Brazil, the loss of transplantable organs from deceased potential donors as a function of cardiac arrest is notably high.

## Objective

To test the hypothesis that a goal-directed protocol to guide the management of deceased donors may reduce the losses of potential donors due to cardiac arrest.

## Methods

Analysis of all deceased donors reported prospectively to CNCDO/SC, over six 4-month periods between May 2012 and April 2014. Hospitals were encouraged to use a checklist to obtain clinical goals during the management of deceased donors. The checklist was composed of the following goals: protocol duration 12-24 hours, temperature >35°C, mean arterial pressure (MAP) >65 mmHg, diuresis 1-4 ml/kg/hour, corticoids, vasopressin if MAP <65 mmHg, tidal volume 6-8 ml/kg, PEEP 8-10 cmH_2_O, sodium <150 mEq/l and blood glucose <180 mg/dl. Effective donors were compared with losses by cardiac arrest. A logistic regression model was used to identify predictors of cardiac arrest with *p *<0.05.

## Results

Of 771 consecutive deceased donors at 27 hospitals, 41 were excluded. Data from the remaining 730 were analyzed. There were 324 (42.0) effective donors, 145 (18.8 %) cardiac arrests, 226 family refusals and 35 contraindications. Compliance with the checklist increased from 52.1 % in the first 4-month period to 85.6 % by the end of 2 years (*p *<0.001). Cardiac arrests decreased from 19.8 % (first 4-month period) to 14.6 % (sixth 4-month period) over 2 years (*p *= 0.26). Comparing the sixth 4-month period with the period before the start of the study (26.4 %), there was statistical significance in the latter (*p *= 0.002), with maintenance of this performance in the two following 4-month periods (Quad 7: 13.8 % and Quad 8: 12.1 %; *p *<0.001). Factors associated with cardiac arrest reduction were: use of the checklist (OR 0.27, 95 % CI 0.16-0.44, *p *<0.001) and temperature >35°C (0.79, 95 % CI 0.19-0.79, *p *= 0.006). The occurrence of cardiac arrests were inversely proportional to the number of interventions (no checklist: 56 %, 0-1: 35 %, 2: 46 %, 3: 38.7 %, 4: 20.2 %, 5: 17.4 %, 6: 12 %, 7: 11 %). More than four interventions had less association with cardiac arrests (30.9 % vs.15.4 %, OR: 0.40, 95 % CI 0.24-0.69, *p *<0.001).

## Conclusion

Meeting clinical goals during the management of deceased donors might reduce the loss of organ donors owing to cardiac arrest.

